# Health-Related Quality of Life in Well-treated People With HIV and Comparators Over an 8-year Period: A Longitudinal Analysis From the Prospective AGE_h_IV Cohort Study

**DOI:** 10.1093/cid/ciag281

**Published:** 2026-05-14

**Authors:** Kevin Moody, Manon C Vanbellinghen, Pythia T Nieuwkerk, Anders Boyd, Maarten F Schim van der Loeff, Suzanne E Geerlings, Peter Reiss, Marc van der Valk, the AGEhIV study group, P Reiss, F W N M Wit, M van der Valk, A Boyd, M L Verburgh, I A J van der Wulp, M C Vanbellinghen, C J van Eeden, M F Schim van der Loeff, J C D Koole, L del Grande, I Agard, S Zaheri, M M J Hillebregt, Y M C Ruijs, D P Benschop, A el Berkaoui, A Boyd, F W N M Wit, N A Kootstra, A M Harskamp-Holwerda, I Maurer, M M Mangas Ruiz, B D N Boeser-Nunnink, O S Starozhitskaya, L van der Hoek, M Bakker, M J van Gils, L Dol, G Rongen, S E Geerlings, A Goorhuis, J W R Hovius, F J B Nellen, J M Prins, T van der Poll, M van der Valk, W J Wiersinga, M van Vugt, G de Bree, B A Lemkes, V Spoorenberg, F W N M Wit, J van Eden, F J J Pijnappel, A Weijsenfeld, S Smalhout, I J Hylkema, van den Bout, C Bruins, M E Spelbrink, P G Postema, P H L T Bisschop, E Dekker, N van der Velde, R Franssen, J M R Willemsen, L Vogt, P Portegies, G J Geurtsen, I Visser, A Schadé, P T Nieuwkerk, R P van Steenwijk, R E Jonkers, C B L M Majoie, M W A Caan, B J H van den Born, E S G Stroes, S van Oorspronk

**Affiliations:** Amsterdam UMC, University of Amsterdam, Infectious Diseases, Amsterdam, The Netherlands; Amsterdam Institute for Immunology and Infectious Diseases (AII), Amsterdam UMC, Amsterdam, The Netherlands; Amsterdam Public Health Research Institute, Amsterdam UMC, Amsterdam, The Netherlands; Amsterdam UMC, University of Amsterdam, Infectious Diseases, Amsterdam, The Netherlands; Amsterdam Institute for Immunology and Infectious Diseases (AII), Amsterdam UMC, Amsterdam, The Netherlands; Amsterdam Public Health Research Institute, Amsterdam UMC, Amsterdam, The Netherlands; Amsterdam Institute for Immunology and Infectious Diseases (AII), Amsterdam UMC, Amsterdam, The Netherlands; Amsterdam Public Health Research Institute, Amsterdam UMC, Amsterdam, The Netherlands; Amsterdam UMC, University of Amsterdam, Medical Psychology, Amsterdam, The Netherlands; Amsterdam UMC, University of Amsterdam, Infectious Diseases, Amsterdam, The Netherlands; Amsterdam Institute for Immunology and Infectious Diseases (AII), Amsterdam UMC, Amsterdam, The Netherlands; Stichting HIV Monitoring, Amsterdam, The Netherlands; Amsterdam Institute for Immunology and Infectious Diseases (AII), Amsterdam UMC, Amsterdam, The Netherlands; Amsterdam Public Health Research Institute, Amsterdam UMC, Amsterdam, The Netherlands; Public Health Service of Amsterdam, Department of Infectious Diseases, Amsterdam, The Netherlands; Amsterdam UMC, University of Amsterdam, Infectious Diseases, Amsterdam, The Netherlands; Amsterdam Institute for Immunology and Infectious Diseases (AII), Amsterdam UMC, Amsterdam, The Netherlands; Amsterdam Public Health Research Institute, Amsterdam UMC, Amsterdam, The Netherlands; Amsterdam Institute for Immunology and Infectious Diseases (AII), Amsterdam UMC, Amsterdam, The Netherlands; Amsterdam Public Health Research Institute, Amsterdam UMC, Amsterdam, The Netherlands; Amsterdam UMC, University of Amsterdam, Global Health, Amsterdam, The Netherlands; Amsterdam Institute for Global Health and Development, Amsterdam UMC, Amsterdam, The Netherlands; Amsterdam UMC, University of Amsterdam, Infectious Diseases, Amsterdam, The Netherlands; Amsterdam Institute for Immunology and Infectious Diseases (AII), Amsterdam UMC, Amsterdam, The Netherlands; Amsterdam Public Health Research Institute, Amsterdam UMC, Amsterdam, The Netherlands; Stichting HIV Monitoring, Amsterdam, The Netherlands

**Keywords:** HIV, aging, health-related quality of life, longitudinal, depression

## Abstract

**Background:**

People with human immunodeficiency virus (HIV) experience more comorbidities, which can reduce health-related quality of life (HRQoL). We compared HRQoL and depressive symptoms over 8 years in people with and without HIV in the AGE_h_IV study.

**Methods:**

We assessed HRQoL biennially from 2010 to 2020 using 36-item Short Form Survey (SF-36) Physical and Mental Component Scores (PCS and MCS) and measured depressive symptoms with the Center for Epidemiologic Studies Depression Scale (CES-D). Participants with ≥1 SF-36 were included; people with HIV not on antiretroviral therapy (ART) or with HIV-1 ribonucleic acid (RNA) > 200 copies/mL at inclusion were excluded. Mean PCS and MCS were compared between groups using mixed-effects linear regression adjusted for sociodemographics, lifestyle, and comorbidity count, with an HIV status × visit interaction. Clinically relevant depressive symptoms (CES-D ≥ 16) were analyzed using mixed-effects logistic regression.

**Results:**

We included 522 people with HIV and 532 comparators, predominantly Dutch men who have sex with men, median ages of 52.9 and 52.1 years, respectively. Over a median 8.0-year follow-up, mean PCS and MCS scores were lower in people with HIV by −1.69 (95% CI, −2.28 to −1.10) and −1.25 (95% CI, −1.97 to −0.53). Clinically relevant depressive symptoms were more frequent in people with HIV (adjusted odds ratio: 2.74; 95% CI, 1.40–5.37). Changes in all endpoints over time did not differ between groups.

**Conclusions:**

Well-treated, middle-aged people with HIV may retain HRQoL comparable to individuals without HIV, with differences that, although statistically significant, might be of unclear clinical relevance due to small effect sizes. Changes in HRQoL and depressive symptoms over time do not differ between groups.

## Graphical Abstract

**Graphical Abstract:**
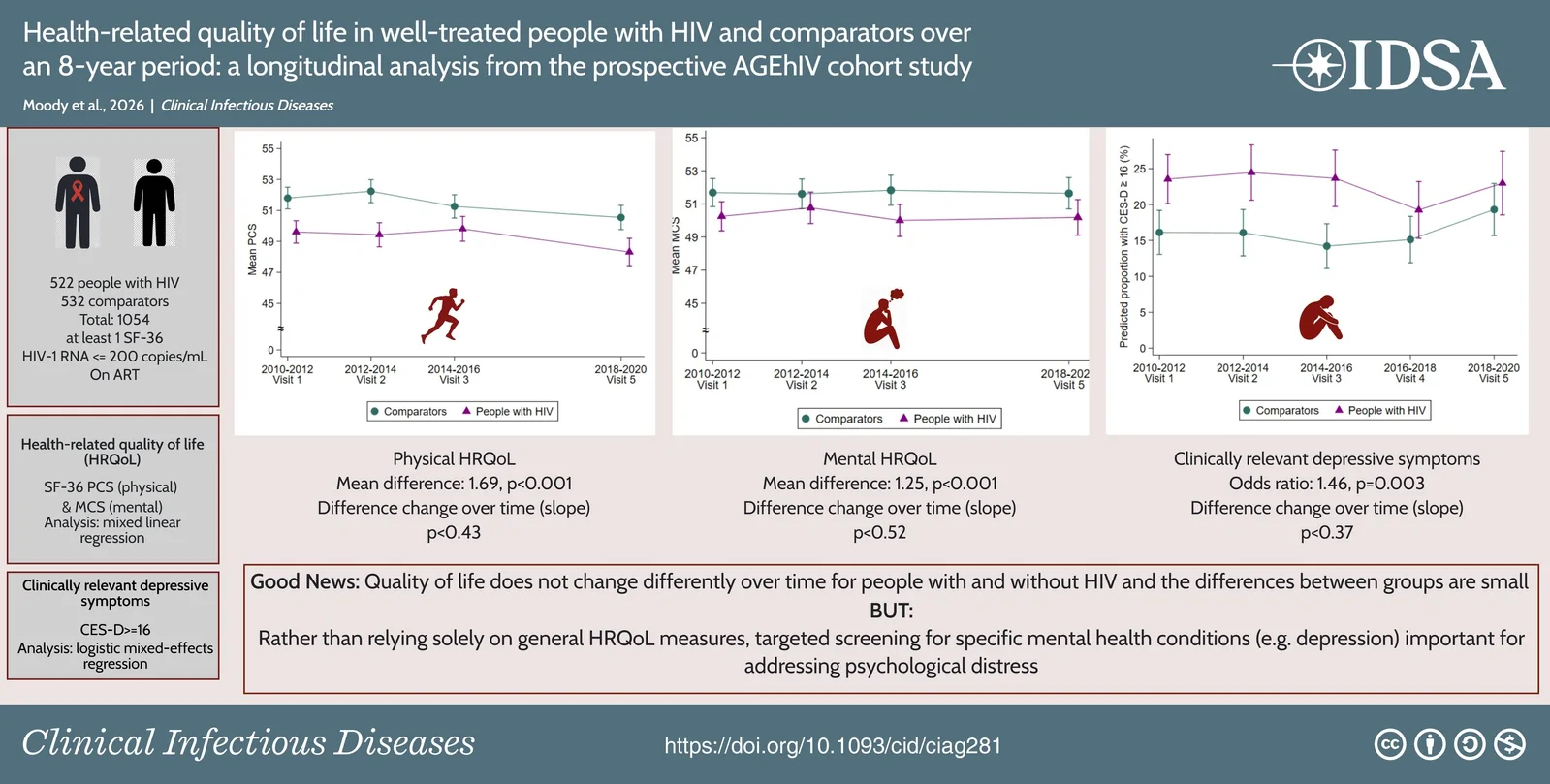
This graphical abstract is also available at Tidbit: https://tidbitapp.io/tidbits/health-related-quality-of-life-in-well-treated-people-with-hiv-and-comparators-over-an-8-year-period-a-longitudinal-analysis-from-the-prospective-agehiv-cohort-study-a783bd9a-3f26-4b06-a58b-a4a719fef7f9/update

## Teaser

Health-related quality of life (HRQoL) is vital in human immunodeficiency virus (HIV) research and care, because viral suppression alone does not define long-term wellbeing. Over 8 years, people with HIV showed persistently modestly lower health-related quality of life (HRQoL), yet changes over time paralleled those without HIV.

**(See the Editorial Commentary by Sabin on pages e368–9.)**

Human immunodeficiency virus type 1 (HIV-1) infection has become a chronic condition, and people with HIV with suppressed viral loads who are taking antiretroviral medicines have a life expectancy approaching that of people without HIV [1]. The biomedical goal of suppressing HIV through antiretroviral treatment and monitoring age-related comorbidities has been joined by calls in recent years for the inclusion of health-related quality of life (HRQoL) as an important additional clinical outcome [2–4].

People with HIV compared to people without HIV experience a higher prevalence of multiple chronic comorbidities, including depression, which, compounded by stigma and discrimination, can significantly impair their HRQoL [5–10]. Depression and HRQoL affect each other bidirectionally with depression predicting a low HRQoL, and low quality of life an impaired mental health status [11]. In cross-sectional studies, having HIV is independently associated with diminished physical and mental HRQoL, and aging people with HIV demonstrate a higher prevalence of depressive symptoms relative to comparable individuals without HIV [12–15]. Additionally, even when compared to individuals with other chronic conditions, including diabetes mellitus (DM) and rheumatoid arthritis, several studies have reported lower mental HRQoL scores in people with HIV [16, 17]. A 3-year longitudinal study looking at the impact of stress on HRQoL undertook separate analyses for people with and without HIV but did not compare differences in changes over time. They found significant but minimal decreases in mental and physical HRQoL over time in people with HIV, but not in people without HIV [18].

We hypothesized that people with HIV may experience lower HRQoL scores and a higher prevalence of depressive symptoms than comparators. We further hypothesized that people with HIV may experience a more rapid decline in HRQoL and proportionately increasing depressive symptoms as they age than people without HIV. We therefore conducted a longitudinal analysis of HRQoL and depressive symptoms over an 8-year period comparing well-treated, aging people with HIV with highly comparable people without HIV, examining both overall group differences and changes over time.

## METHODS

### Study Population

The AGE_h_IV Cohort Study is an ongoing prospective observational cohort study assessing the prevalence and incidence of age-related comorbid conditions and their risk factors in participants with and without HIV aged ≥45 years. Between 2010 and 2012, people with HIV were recruited at the outpatient HIV clinic of the Amsterdam University Medical Center (UMC) and participants without HIV from either the sexual health clinic or the Amsterdam Cohort Studies on HIV/AIDS at the Public Health Service Amsterdam. At baseline and every 2 years thereafter, all participants undergo standardized screening for age-associated comorbidities, which includes completing a standardized questionnaire. Details have been described previously [19].

For the current analysis, we included participants who completed at least one 36-item Short Form Survey (SF-36) at 1 or more of study visits 1 (2010–2012), 2 (2012–2014), 3 (2014–2016), or 5 (2018–2020). To reduce potential variation in scores of HRQoL attributed to individuals without HIV virological control, we excluded participants with HIV who were not on antiretroviral therapy (ART) or on ART with HIV-1 ribonucleic acid (RNA) > 200 copies/mL at study enrollment.

### Data Collection and Study Variables

We recorded data on age, sex at birth, origin (Dutch, defined as born in the Netherlands with both parents also born in the Netherlands, vs non-Dutch), relationship status (categorized as married/cohabitating or not), and educational level (categorized as high—ie bachelor's degree or higher—or not) at baseline, defined as the date of the first SF-36 completed by participants. We collected data on physical activity, alcohol consumption, current tobacco use, use of tetrahydrocannabinol (THC), and use of 3,4-methylenedioxymethamphetamine (MDMA) at each study visit. We defined people as being physically active when they performed moderate physical activity ≥5 days per week for ≥30 minutes, or when they performed heavy physical activity at least twice a week for ≥20 minutes. We considered heavy daily alcohol consumption as >4 IU/day for men and >2 IU/day for women daily or almost daily. We categorized the use of THC as daily to monthly use versus less often, and the use of MDMA as daily to monthly versus less often. We collected data on comorbidities, including DM, non-AIDS defining malignancies, osteoporosis, chronic kidney disease, cardiovascular disease, chronic obstructive pulmonary disease (COPD), and obesity at each visit. Definitions of these comorbidities are provided in Supplementary Table 1. We counted and categorized the number of comorbidities as 0, 1, 2, or 3 or more. We asked participants to complete the questionnaire Center for Epidemiologic Studies Depression Scale (CES-D) at all visits and the SF-36 at all study visits except for visit 4. Starting at visit 4, a change in protocol was implemented to alternate between the SF-36 and the EuroQol five-Dimensional Questionnaire (EQ-5D), which reduced questionnaire burden because of fewer questions. For people with HIV, we collected the date of HIV diagnosis, CD4 nadir and prior documented AIDS events at enrollment, and data on plasma HIV-1 RNA, CD4+ and CD8+ cell counts, at enrollment and each subsequent visit.

### Definitions of Outcomes

Physical and mental HRQoL were assessed using the SF-36. Physical and Mental Component Scores (PCS and MCS) were calculated according to the standard scoring manual [20]. We transformed individual item responses to the SF-36 into 8 subscale scores ranging from 0 to 100. We then calculated Z-scores for these 8 subscales using the mean and SDs from the general US population. These Z-scores were then combined by applying specific factor loadings to calculate the raw PCS and MCS, which were then standardized to T-scores with a population mean of 50 and SD of 10 [21]. For both PCS and MCS, higher scores indicate better self-reported physical or mental health. There is no cutoff to determine a specific metric of high or low quality of life. We determined the presence of clinically significant depressive symptoms by applying a cutoff score of ≥16 to the CES-D as an additional outcome variable [22].

### Statistical Analysis

We compared sociodemographic and lifestyle characteristics at enrollment between people with and without HIV using Pearson χ^2^, Fisher’s exact test, or Mann–Whitney *U* tests, as appropriate.

We used mixed linear regression to model individual PCS and MCS, with visit number, HIV status, and their interaction as covariates to assess differences in changes over time. Estimated mean PCS and MCS were then derived from this model. We included a random intercept to account for between-participant variability at inclusion and repeated measurements within participants. We determined the odds of having clinically significant symptoms of depression (yes/no) and the differences in changes in odds ratios between people with HIV and comparators over time using logistic mixed-effects regression with a random intercept and random slope, again with the covariates visit number, HIV status, and the interaction between study visit and HIV status. We report the number and percentage of completed SF-36 and CES-D questionnaires included in the analyses at each visit.

We initially built our multivariable model 1 by adjusting a priori for age, sex at birth, origin, marital status, educational level, and physical activity. We included physical activity in the first model to minimize bias due to confounding when comparing HRQoL between groups. To evaluate the sensitivity of our findings to this modeling choice, we compared estimates with and without adjustment for physical activity. If the change in the coefficient was less than 10%, this would suggest minimal mediation from physical activity in the relationship between HIV status and HRQoL. We then added the substance use covariances, alcohol use, current tobacco use, THC use, and MDMA use, one by one to this model, retaining the covariate if it resulted in improved model fit, as indicated by a lower Bayesian information criterion (BIC). Finally, in model 2, we included the number of comorbidities to model 1, without regard to its impact on model fit.

Prior to analyzing all models, we tested collinearity by calculating the variance inflation factor (VIF) from a linear regression model using all covariables and our independent variables; those with a VIF above 5 were removed from the model. Statistical analyses were carried out using SPSS software (version 28) and STATA software (version 17.1; StataCorp). Statistical significance was defined as a 2-sided *P* value of <.05. We reported our findings using the STrengthening the Reporting of OBservational studies in Epidemiology (STROBE) guidelines for cohort studies [23].

### Role of the Funding Source

The funders of the study had no role in study design, data collection, data analysis, data interpretation, or writing of the report.

## RESULTS

### Participant Characteristics at Inclusion

**Figure 1.**
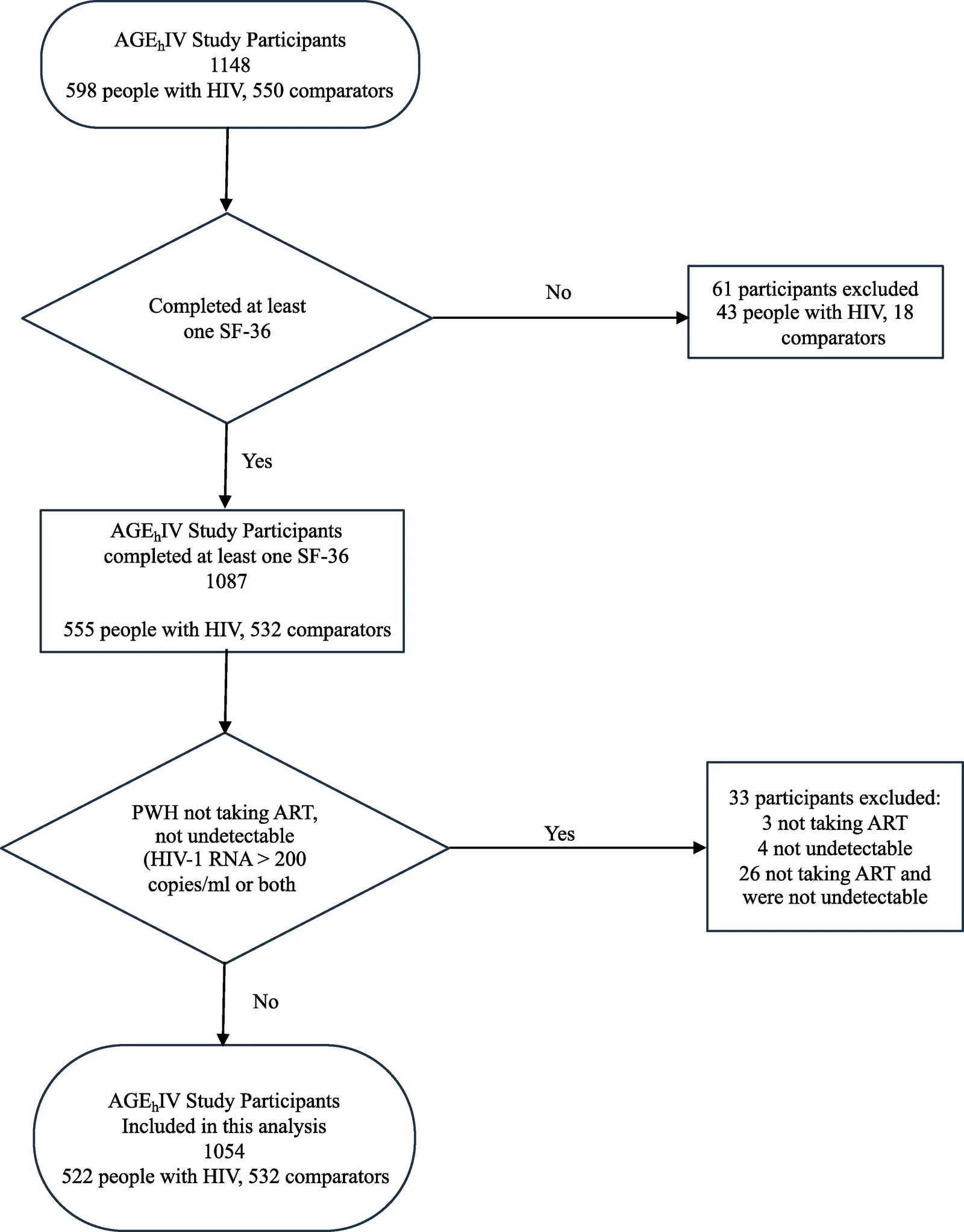
Inclusion of participants into this analysis from the AGE_h_IV cohort from 2010 to 2020 in Amsterdam, the Netherlands. Abbreviations: ART, antiretroviral therapy; HIV, human immunodeficiency virus; PWH, people with HIV; RNA, ribonucleic acid; SF-36, 36-item Short Form Survey.

A total of 1148 participants were eligible for inclusion. After excluding those without any completed SF-36 questionnaire and people with HIV not on ART or whose HIV-1 RNA was >200 copies/mL, 522 people with HIV and 532 comparators remained in this analysis (Figure 1). The median number of visits was 3 (interquartile range [IQR]: 2–4) for people with HIV and 4 (IQR: 3–4) for comparators. The median follow-up of participants was 8.0 years (IQR 4.4–8.3) for people with HIV and 8.1 (IQR 7.9–8.4) for comparators.

The characteristics at enrollment of participants, including their baseline PCS, MCS, and prevalence of clinically relevant depressive symptom scores, are shown in Table 1. In people with HIV, there were more men, fewer Dutch people, more who were married or cohabitating, and fewer with higher education than in comparators. Compared to comparators, they were less physically active, less likely to engage in heavy alcohol consumption, more likely to be current smokers, and less likely to use MDMA daily to monthly. They had a significantly higher number of comorbid conditions compared to comparators. People with HIV had been diagnosed with HIV a median of 12.7 years (IQR: 7.2–17.5) at enrollment, and one-third had experienced a prior AIDS event.

**Table 1.** Characteristics of AGE_h_IV Cohort Study, Amsterdam, the Netherlands: People With HIV (n = 522) and Comparators (n = 532) at the Time of Enrollment

	People With HIV	Comparators	*P* Value
**Total, n**	522	532	
Demographic			
Age (y)	52.9 (48.2–59.6)	52.1 (47.9–58.4)	.16^a^
Male sex at birth	467 (89.5)	449 (84.4)	.017^c^
Dutch	367 (72.8)	427 (81.3)	<.001^c^
MSM	398 (76.7)	369 (69.9)	.014^c^
Married/cohabitating status	252 (50.1)	220 (41.9)	.009^c^
High education level^d^	208 (41.4)	289 (55.8)	<.001^c^
Lifestyle/substances			
Physically active^e^	219 (43.7)	275 (52.7)	.005^c^
Alcohol use, heavy^f^	19 (3.6)	35 (6.6)	.036^c^
Tobacco use, current	176 (33.7)	133 (25.0)	.002^c^
THC use, daily to monthly	70 (13.5)	57 (10.8)	.19^c^
MDMA use, daily to monthly	23 (4.4)	47 (8.9)	.004^c^
Clinical			
Number of comorbidities			
0	244 (46.8)	329 (61.8)	<.001^b^
1	168 (32.2)	161 (30.3)	
2	70 (13.4)	26 (4.9)	
≥3	39 (7.5)	16 (3.0)	
HIV related			
Time since HIV diagnosis (y)	12.7 (7.2–17.5)	…	
HIV-1 RNA ≤ 200 copies/mL	522 (100)	…	
CD4 count (cells/µL)	570 (450–770)	…	
CD8 count (cells/µL)	840 (610–1130)	…	
Nadir CD4 (cells/µL)	170 (70–250)	…	
Prior AIDS event	174 (33.3)	…	
Dependent variables			
PCS	51.6 (44.2–55.7)	54.3 (49.6–57.1)	<.001^a^
MCS	53.4 (44.9–57.9)	54.7 (47.1–58.3)	.032^a^
CES-D	7.0 (2.0–15.0)	4.0 (1.0–11.0)	<.001^a^
CES-D score ≥ 16	119 (23.8)	85 (16.3)	.003^c^

Data are presented as n (%) or median (IQR).

Abbreviations: CD4, cluster of differentiation 4; CD8, cluster of differentiation 8; CES-D, Center for Epidemiologic Studies Depression Scale; HIV, human immunodeficiency virus; MCS, Mental Component Score of the SF-36; MDMA, 3,4-methylenedioxymethamphetamine; MSM, men who have sex with men; PCS, Physical Component Score of the 36-item Short Form Survey (SF-36); THC, tetrahydrocannabinol.

^a^Mann–Whitney *U* test.

^b^Pearson chi-squared test.

^c^Fisher's exact test.

^d^High education level defined as attainment of bachelor's degree or higher.

^e^Physically active defined as moderate physical activity ≥5 d per week for ≥30 m, or heavy physical activity at least twice a week for ≥20 m.

^f^Heavy alcohol use defined as >4 units per day for men and >2 units per day for women.

The median PCS at enrollment was 51.6 (IQR: 44.2–55.7) for people with HIV and 54.3 (IQR: 49.6–57.1) for comparators (Table 1). For MCS, this was 53.4 (IQR: 44.9–57.9) and 54.7 (IQR: 47.1–58.3), respectively. For CES-D, the median score at enrollment was 7 (IQR: 2–15) for people with HIV and 4 (IQR: 2–15) for comparators, while the percentage of people with clinically depressive symptoms (CES-D ≥ 16) was 23.8% for people with HIV and 16.3% for comparators.

### Physical Component Score

**Figure 2.**
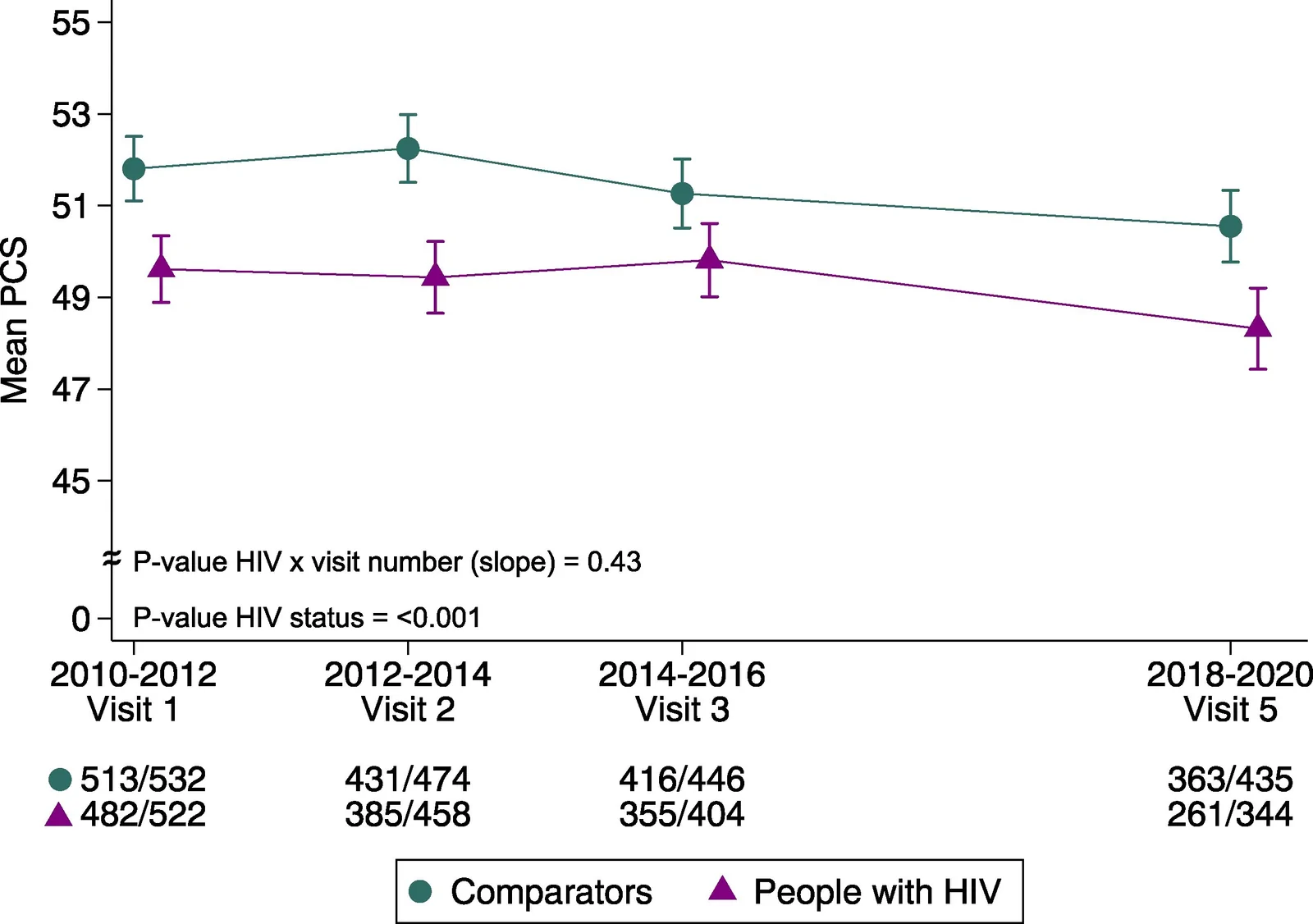
Comparison of PCS scores from 2010 to 2020 in AGE_h_IV Cohort people with HIV and comparators, Amsterdam, the Netherlands. The figure shows the PCS mean (in dots/triangles) and 95% CIs (error bars) from the mixed linear regression model for model 2, which includes the number of comorbidities. Numbers of questionnaires included (numerator) and participants in active follow-up (denominator) at each timepoint appear below each visit. Abbreviations: CI, confidence interval; HIV, human immunodeficiency virus; PCS, Physical Component Score summary of the 36-item Short Form Survey (SF-36).

For PCS over time in people with HIV and comparators, all substance use covariates were retained in the final model 1 because they all contributed to improved model fit. People with HIV had significantly and consistently lower mean PCS (model 1: −2.25 [95% CI, −2.84 to −1.66]; *P* < .0001; Supplementary Figure 1); this difference was somewhat attenuated when including the number of comorbidities (model 2: −1.69 [95% CI, −2.28 to −1.10]; *P* < .0001; Figure 2). There was no difference in the change over time in both models for mean PCS between people with HIV and comparators (model 1, *P* for interaction = .57; model 2, *P* for interaction = .43).

### Mental Component Score

**Figure 3.**
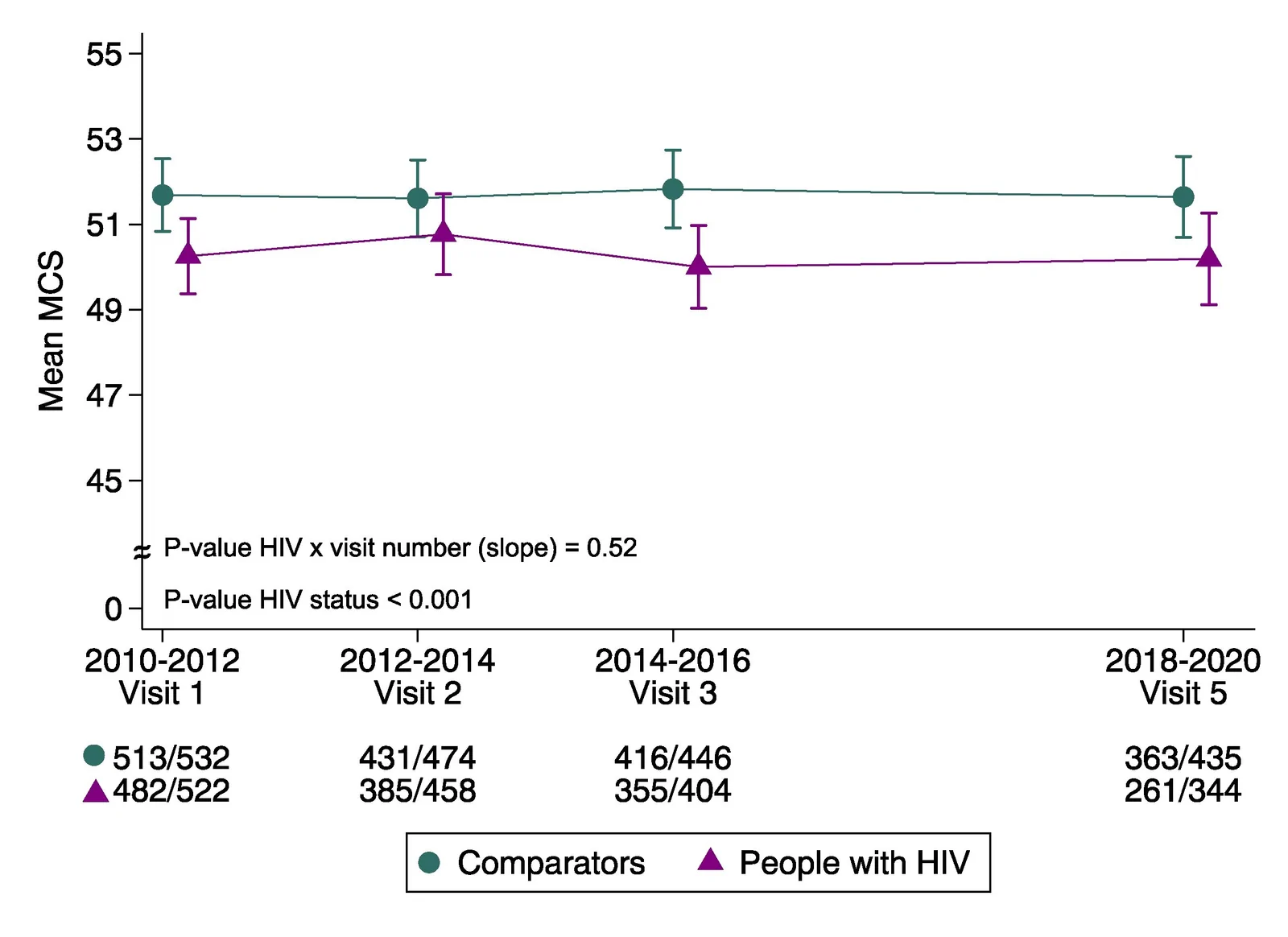
Comparison of MCS scores from 2010 to 2020 in AGE_h_IV Cohort people with HIV and comparators Amsterdam, the Netherlands. The figure shows the MCS mean (in dots/triangles), stratified by HIV status, and 95% CIs (in bars) for model 2, which includes the number of comorbidities. Numbers of questionnaires included (numerator) and participants in active follow-up (denominator) at each timepoint appear below each visit. Abbreviations: CI, confidence interval; HIV, human immunodeficiency virus; MCS, Mental Component Score summary of the 36-item Short Form Survey (SF-36).

For MCS over time, all substance use covariates were retained in the final models because they all contributed to improved model fit. The mean MCS were significantly lower in people with HIV compared to comparators (model 1: −1.27 [95% CI, −1.97 to −0.57]; *P* < .0001; Supplementary Figure 2), which remained so when adding total comorbidities (model 2: −1.25 [95% CI, −1.97 to −0.53]; *P* < .0001; Figure 3). There was no difference in the changes over time in both models for mean MCS between people with HIV and comparators over time (model 1, *P* = .50; model 2, *P* = .52).

### Center for Epidemiologic Studies Depression Scale

**Figure 4.**
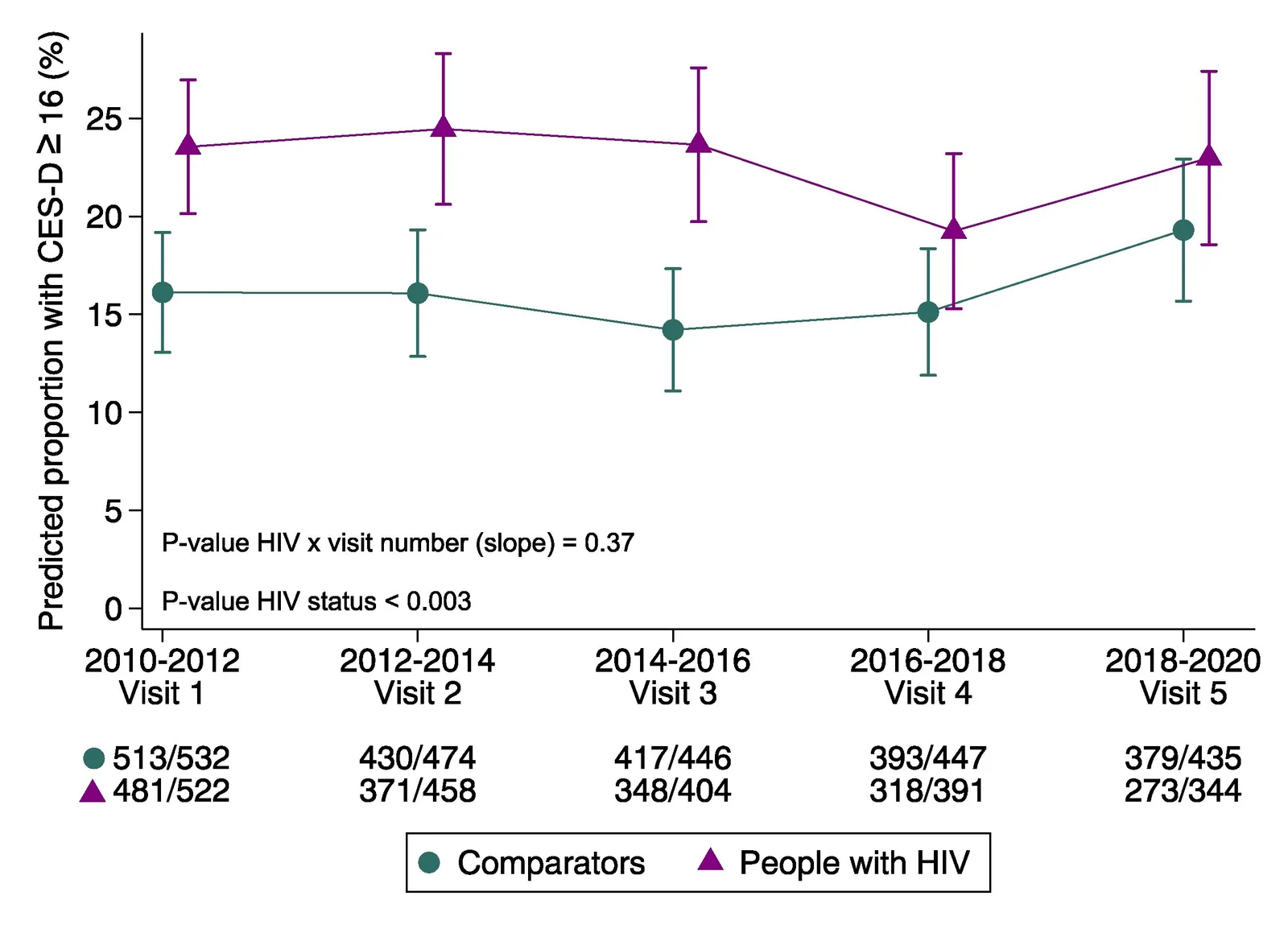
Comparison of predicted proportion of participants with CES-D scores ≥ 16 from 2010 to 2020 in AGE_h_IV Cohort people with HIV and comparators, Amsterdam, the Netherlands. The figure shows the predicted proportion as a percentage (in dots/triangles) of people with clinically relevant depressive symptoms, stratified by HIV status, 95% CIs (in bars) from model 2, which includes the number of comorbidities. Numbers of questionnaires included (numerator) and participants in active follow-up (denominator) at each timepoint appear below each visit. Abbreviations: CES-D, Center for Epidemiologic Studies Depression Scale; CI, confidence interval; HIV, human immunodeficiency virus.

For the proportion of individuals reporting clinically relevant depressive symptoms over time with the CES-D, only THC and MDMA contributed to improved model fit and were added to the final models. People with HIV had higher odds of clinically relevant depressive symptoms than comparators over the period (model 1: 2.90 [95% CI, 1.50–5.63]; *P* = .002; Supplementary Figure 3), which remained when comorbidities were added to the model (model 2: 2.74 [95% CI, 1.40–5.37]; *P* = .003; Figure 4). There was no difference in changes over time overall in the proportion with depressive symptoms between people with HIV and comparators (model 1, *P* = .30; model 2, *P* = .37).

## DISCUSSION

This is the first longitudinal study comparing aging people with HIV to demographic- and lifestyle-similar comparators for physical and mental HRQoL. People with HIV experienced lower physical and mental HRQoL scores over the period, while changes over time in these domains were similar during a median of around 8 years of follow-up. While the statistically significant lower HRQoL scores among people with HIV are consistent with our hypothesis, the differences observed are of uncertain clinical significance. The results contrast with our second hypothesis that HRQoL would decline more rapidly with age in people with HIV.

Human immunodeficiency virus has long been hypothesized to influence the aging process, either through accentuated aging, reflecting an additional baseline risk associated with HIV infection, or accelerated aging, defined as a steeper increase in risk over time [9]. While some studies have suggested biological mechanisms that could support accelerated aging, longitudinal evidence remains limited [24–26]. In our study, the trajectories of physical and mental health–related quality of life over time are compatible with accentuated aging but do not provide evidence for accelerated aging. This interpretation is consistent with earlier findings from our cohort showing no acceleration in the long-term evolution of physical comorbidities in people with HIV on effective ART [6].

Human immunodeficiency virus was a significant independent predictor for lower PCS scores throughout the duration of the study. Adjusting for comorbidities in the model partially attenuated the observed difference in PCS between people with HIV and comparators, aligning with findings in a previous cross-sectional study [12]. This is likely explained by comorbidities that are more common in people with HIV, adversely affecting physical HRQoL. The difference in mean PCS was only 2 points on a 100-point scale, which may not be clinically relevant. Nevertheless, the persistence of the difference in PCS—even after accounting for comorbidities—suggests that other factors related to HIV burden may contribute to lower perceived physical HRQoL in people with HIV, such as HIV-related stigma and/or issues related to differences in socioeconomic status (eg income, education, and housing) [8, 27, 28].

The mean mental HRQoL was also somewhat lower throughout the duration of the study in people with HIV compared to comparators, which is consistent with findings reported elsewhere [12, 29, 30]. However, the difference in MCS between people with HIV and comparators was minimal (approximately 1 point on a 100-point scale) and perhaps not clinically relevant.

Despite the small difference in mean MCS between people with HIV and comparators, we found that people with HIV had significantly higher odds of experiencing clinically relevant depressive symptoms compared to comparators. The number of comorbidities did not impact the observed association. This analysis confirms earlier cross-sectional analysis from both our cohort [12] and another study showing higher probability of depressive symptoms in people with HIV [31]. The absence of clinically meaningful differences in mental HRQoL as measured by the MCS contrasted with the higher prevalence of depressive symptoms detected by the CES-D. These results suggest that measuring the broader and more general construct of mental health did not detect any major differences between people with HIV and comparators, whereas differences were found when the narrower construct of depressive symptoms was measured. This underscores the importance of supplementing instruments that measure broad constructs, such as HRQL, with instruments that more specifically measure expected relevant outcomes in a particular population [32]. In this way, different dimensions of psychological wellbeing may be better captured, which is particularly important given the higher incidence of issues, such as depression in people with HIV, than in the general population and, specifically in gay men, who made up the bulk of our population [10, 33]. Like with the physical and mental HRQoL, changes over time in clinically relevant depressive symptoms in people with HIV did not differ from those in comparators.

An important strength of our study is its longitudinal design, which provided us with the opportunity to analyze differences over an extended period between well-treated people with HIV and comparators. In addition, participants in both groups were recruited from care settings that serve similar populations, contributing to a broadly comparable study population, and all underwent identical assessments. We found statistically significant differences in baseline characteristics between people with HIV and comparators. Given that the absolute differences are small, it is unclear whether they would be clinically relevant. We accounted for these differences by adjusting for them in our models. In terms of limitations, the CES-D is a self-reported screening tool for depressive symptoms and does not equate a clinical diagnosis; thus, findings should be interpreted as indicative rather than definitive. As with most cohorts, ours experienced attrition over time. To address missing data, we used linear and logistic mixed-effects models. These models employ likelihood-based estimation, which yields unbiased parameter estimates under the assumption that the data are missing at random (MAR). To assess the validity of this assumption, we examined whether the probability of attrition was related to observed health outcomes. Our analysis indicated that participants with lower mental health scores at earlier visits had significantly fewer subsequent follow-up visits. This finding supports the MAR assumption, as it demonstrates that the missingness mechanism is conditionally dependent on observed data, and under this assumption, the models yield unbiased estimates. Finally, it should be noted that our results apply to older, predominantly white people with HIV in an urban setting with good access to healthcare and, for people with HIV, well-treated HIV infection.

## CONCLUSIONS

Although mean HRQoL scores were significantly lower among people with HIV, the differences were minimal and of uncertain clinical relevance. However, people with HIV had almost 3 times higher odds of reporting clinically relevant depressive symptoms. In terms of changes across timepoints, participants with and without HIV over a median of 8 years showed no significant differences in the changes of physical or mental HRQoL, nor in changes over time in the odds of experiencing depressive symptoms. Overall, our findings suggest that targeted screening for specific mental health conditions may be more informative for identifying and addressing psychological distress in people with HIV than relying solely on general HRQoL measures.

## Supplementary Material

ciag281_Supplementary_Data

## Data Availability

Data sharing has been restricted by the Ethical Review Board of the Amsterdam University Medical Centers because the data underlying this study contain sensitive and potentially identifying information. Requests for data sharing can be made via submission of a concept sheet as per instructions on the project website (https://agehiv.nl/en/science/). Once submitted, the proposed research or analysis will undergo review for evaluation of the scientific value, relevance to the study, design and feasibility, statistical power, and overlap with existing projects. If the proposed analysis is for verification or replication, data will then be made available. If the proposed research is for novel science, feedback will be provided to the proposers upon completion of the review. In some circumstances, a revision of the concept can be requested. If the concept is approved for implementation, a writing group will be established consisting of the proposers (ie, up to three individuals that were centrally involved in the development of the concept) and members of the AGEhIV Cohort Study group or other appointed cohort representatives. All individuals involved in the process of reviewing these research concepts are bound by confidentiality. Deidentified participant data, along with a data dictionary, will be made available if the concept is approved. Study protocol and blank informed consent forms can also be provided on request.

## Supplementary Data

Supplementary materials are available at *Clinical Infectious Diseases* online. Consisting of data provided by the authors to benefit the reader, the posted materials are not copyedited and are the sole responsibility of the authors, so questions or comments should be addressed to the corresponding author.
