# Supplementary material for: Health-Related Quality of Life in Well-treated People With HIV and Comparators Over an 8-year Period: A Longitudinal Analysis From the Prospective AGEhIV Cohort Study
Source: Clin Infect Dis. 2026 May 14;83(2):e357–67. doi: 10.1093/cid/ciag281 (PMC13537125; doi:10.1093/cid/ciag281)
Supplement: Supplementary file 1 [file ciag281_supplementary_data.docx]

**Supplementary Material for Health-related quality of life and depression over time in ageing people with HIV is not different to that of well-matched comparators without HIV: an 8-year longitudinal analysis from the AGE_h_IV cohort study**

**Table of contents**

Supplementary Table 1: Definition of comorbidities for AGE_h_IV Cohort Study comparison of health related quality of life from 2010-2020, Amsterdam, the Netherlands

Supplementary Figure 1: Comparison of PCS scores from 2010 to 2020 in AGE_h_IV Cohort people with HIV and comparators, Amsterdam, the Netherlands.

Supplementary Figure 2: Comparison of MCS scores from 2010 to 2020 in AGE_hI_V Cohort people with HIV and comparators, Amsterdam, the Netherlands.

Supplementary Figure 3: Comparison of predicted proportion of participants with CES-D scores ≥ 16 from 2010 to 2020 in AGE_h_IV Cohort people with HIV and comparators, Amsterdam, the Netherlands.

Supplementary Table 1: Definition of comorbidities for AGE_h_IV Cohort Study comparison of health related quality of life from 2010-2020, Amsterdam, the Netherlands

| **Comorbidity^1^** | **Definition** |
| --- | --- |
| Chronic obstructive pulmonary disease (COPD) or asthma | If 1-second forced expiratory volume (FEV1) to forced vital capacity (FVC) ratio was <0.7 for 3 forced expiratory spirometric measurements (MicroDirect Spiro USB) and having an FEV1 lower than 80% of the predicted value [i.e., Global Initiative for Chronic Obstructive Lung Disease (GOLD) stage ≥2] (https://goldcopd.org) |
| Cardiovascular disease (CVD) | Myocardial infarction (MI), angina pectoris, peripheral artery disease, ischemic stroke, hemorrhagic stroke and heart failure. |
| Chronic kidney disease | An estimated glomerular filtration rate (eGFR) <60 mL/min/1.73 m^2^, as determined by the CKD-EPI equation and/or an albumin/creatinine ratio of ≥3mg/mmol. People with HIV who initiated antiretroviral medication known to inhibit tubular secretion of creatinine (i.e., dolutegravir, bictegravir, rilpivirine and/or cobicistat) prior to meeting these criteria were not classified as having chronic kidney disease. |
| Non-AIDS malignancies | All malignancies, excluding non-melanoma skin cancers and premalignant conditions. In people with HIV, non-Hodgkin lymphoma, cervical cancer and Kaposi sarcoma were categorized as AIDS-associated and excluded. |
| Type 2 Diabetes | Any of the following criteria: HbA1c level of $\geq$48 mmol/mol, non-fasting blood glucose $\geq$11.1 mmol/L, fasting blood glucose $\geq$7.0 mmol/L, the use of antidiabetic medication |
| Osteoporosis | A dual-energy X-ray absorptiometry (DXA) T-score score of $\leq$-2.5 in at least one of three locations (femur, lumbar spine, or hip). For men younger than 50 and premenopausal women a z-score of $\leq$-2 was used according to the recommendations from the International Society for Clinical Densitometry [2]. The DXA results from the third follow-up visit were carried forward to the fourth follow-up visit, as no DXA scans were obtained at this visit. |
| Obesity | Body mass index (BMI) ≥ 30 |

1. Comorbidities reported by participants during study visits were validated using hospital records from the Amsterdam University Medical Centers for people with HIV and medical records from general practitioners (GP) for comparators, provided consent had been provided for contacting the GP. In cases where validation was not possible, self-reported diagnoses were used. Type 2 diabetes, osteoporosis, frailty and kidney disease were considered present if confirmed through study visit assessment. Cardiovascular comorbidities were considered as being present during all study visits following the initial diagnosis, except for MI, which was only counted if it occurred within the 24 months preceding the specific visit. Non-AIDS malignancies were counted as present for 5 years after initial diagnosis; a different primary malignancy was counted separately.

P-value HIV x visit number (slope) = 0.57

P-value HIV status < 0.001

Supplementary Figure 1: Comparison of PCS scores from 2010 to 2020 in AGE_h_IV Cohort people with HIV and comparators, Amsterdam, the Netherlands. The figure shows the PCS mean (in dots/triangles) and 95% confidence intervals (error bars) from the mixed linear regression model for model 1 without comorbidities. Numbers of questionnaires included (numerator) and participants in active follow-up (denominator) at each timepoint appear below each visit. Abbreviations: PCS – Physical component summary of the 36-item Short Form Survey (SF-36); HIV – human immunodeficiency virus.

P-value HIV x visit number (slope) = 0.50

P-value HIV status < 0.001

Supplementary Figure 2: Comparison of MCS scores from 2010 to 2020 in AGE_h_IV Cohort people with HIV and conparators Amsterdam, the Netherlands. The figure shows the MCS mean (in dots/triangles), stratified by HIV status, and 95% confidence intervals (in bars) for model 1 without comorbidities. Numbers of questionnaires included (numerator) and participants in active follow-up (denominator) at each timepoint appear below each visit. Abbreviations: MCS – Mental component summary of the 36-item Short Form Survey (SF-36); HIV – human immunodeficiency virus.

P-value HIV x visit number (slope) = 0.30

P-value HIV status = 0.002

Supplementary Figure 3: Comparison of predicted proportion of participants with CES-D scores ≥ 16 from 2010 to 2020 in AGE_h_IV Cohort people with HIV and comparators, Amsterdam, the Netherlands. The figure shows the predicted proportion as a percentage (in dots/triangles) of people with clinically relevant depressive symptoms, stratified by HIV status, 95% confidence intervals (in bars) from model 1 without comorbidities. Numbers of questionnaires included (numerator) and participants in active follow-up (denominator) at each timepoint appear below each visit. Abbreviations: CES-D – Center for Epidemiologic Studies – Depression Scale; HIV – human immunodeficiency virus.
